# ALE meta-analyses of voxel-based morphometry studies: Parameter validation via large-scale simulations

**DOI:** 10.1016/j.neuroimage.2023.120383

**Published:** 2023-09-20

**Authors:** Lennart Frahm, Theodore D. Satterthwaite, Peter T. Fox, Robert Langner, Simon B. Eickhoff

**Affiliations:** a Department of Psychiatry, Psychotherapy and Psychosomatics, School of Medicine, RWTH Aachen University, Aachen, Germany; b Institute of Neuroscience and Medicine (INM7: Brain and Behaviour), Research Centre Jülich, Jülich, Germany; c Institute of Systems Neuroscience, Medical Faculty, Heinrich Heine University, Düsseldorf, Germany; d Department of Psychiatry, Perelman School of Medicine, University of Pennsylvania, Philadelphia, USA; e Penn Lifespan Informatics and Neuroimaging Center, Perelman School of Medicine, University of Pennsylvania, Philadelphia, USA; f Research Imaging Institute, University of Texas Health Science Center, San Antonio, USA; g Departments of Radiology, Neurology, Psychiatry and Behavioral Sciences, and Physiology, University of Texas Health Science Center, San Antonio, USA

## Abstract

Activation likelihood estimation (ALE) meta-analysis has been applied to structural neuroimaging data since long, but up to now, any systematic assessment of the algorithm’s behavior, power and sensitivity has been based on simulations using functional neuroimaging databases as their foundation. Here, we aimed to determine whether the guidelines offered by previous evaluations can be generalized to ALE meta-analyses of voxel-based morphometry (VBM) studies. We ran 365000 distinct ALE analyses filled with simulated experiments, randomly sampling parameters from BrainMap’s VBM experiment database. We then examined the algorithm’s sensitivity, its susceptibility to spurious convergence, and its susceptibility to excessive contributions by individual experiments. In general, the performance of the ALE algorithm was highly comparable between imaging modalities, with the algorithm’s sensitivity and specificity reaching similar levels with structural data as previously observed with functional data. Because of the lower number of foci reported and the higher number of participants usually included in structural experiments, individual studies had, on average, a higher impact towards significant clusters. To prevent significant clusters from being driven by single experiments, we recommend that researchers include at least 23 experiments in a VBM ALE dataset, instead of the previously recommended minimum of n = 17. While these recommendations do not constitute hard borders, running ALE analyses on smaller datasets would require special diligence in assessing and reporting the contributions of experiments to individual clusters.

## 1. Introduction

Activation Likelihood Estimation (ALE) is a widely used approach for coordinate-based neuroimaging meta-analysis and has been under constant methodological development and review since its inception in 2002 (Acar et al., 2018; Eickhoff et al., 2009, Turkeltaub et al., 2002, 2012). Recent methodological evaluations of ALE have begun to use large-scale empirically informed simulations (Frahm et al. 2022; Eickhoff et al. 2016). While offering valuable insights, the results of such simulations are highly dependent on the data and parameters the simulations are based on. All ALE-related simulation work up to now has used the functional neuroimaging part of the BrainMap database (Fox and Lancaster, 2002; Laird et al., 2005) as the basis for its data creation, but the ALE algorithm is also applied to structural neuroimaging datasets as anatomic likelihood estimation. Notable examples include the 2015 meta-analysis by Goodkind et al. which identified common neurobiological substrates across mental illnesses or Gray and colleagues (2020) paper which highlighted abnormalities in brain structure in major depressive disorder (other examples: Cauda et al., 2018 & Vanasse et al., 2021). The current study aimed to extend previous simulations, leveraging BrainMap’s fairly recently released structural database (Vanasse et al., 2018). This is motivated by the fact that functional imaging experiments differ quite strongly from structural imaging experiments with respect to the average number of participants included and the number of significant coordinates reported (see Tables 1 and 2). Both numbers are parameters of the ALE algorithm and, therefore, can have a major impact on the meta-analytic results, which makes it very important to evaluate the validity of assumptions made by previous simulation studies and the recommendations derived from them, using simulations based on a structural neuroimaging database.

In 2016, Eickhoff et al. investigated the behavior, sensitivity and power of ALE when presented with different thresholding techniques using a simulation setup. Importantly, these simulations were used to estimate a lower bound of experiments researchers should aim to include in their ALE analysis dataset. The basis for this lower bound was formed by power calculations, showing that a dataset should contain at least 15–20 experiments for the algorithm to be able to detect non-trivial convergence. Additionally, this lower bound limits the likelihood of significant clusters being driven by single experiments. The datasets were created by randomly sampling experimental characteristics (number of subjects, number of foci reported) from the functional BrainMap database. As yet, it has remained unclear whether the recommendations made by the paper also hold for other types of neuroimaging data such as those obtained from voxel-based morphometry (VBM).

In this study we aimed to determine whether the guidelines offered by previous studies can be generalized to VBM meta-analyses. To this end, we ran 365000 distinct ALE analyses on datasets filled with simulated experiments, randomly sampling parameters from BrainMap’s structural database. We then evaluated the results obtained with two standard significance thresholding methods, voxel- and cluster-level family-wise error rate (vFWE and cFWE), by examining sensitivity and susceptibility to spurious convergence. Lastly, we assessed ALE’s susceptibility to excessive contributions by individual experiments when dealing with VBM data.

## 2. Methods

Our methodological set-up comprised three distinct steps: (1) simulating datasets based on parameter distributions derived from the VBM BrainMap database, (2) calculating an ALE analysis for each dataset, (3) evaluating the results and the algorithm’s behavior across the whole parameter range on different outcome measures. It should be noted that for each dataset we systematically manipulated the number of truly converging foci, which made it possible to assess power and sensitivity at different levels of evidence strength.

### 2.1. Simulated datasets

To cover the large range of possible dataset sizes encountered in naturalistic research, we simulated and analyzed datasets containing between 15 and 45 experiments in total. While the number of participants in VBM studies is generally higher than in studies using task-related functional imaging, the total number of experiments available for meta-analysis per domain is not noticeably different. This is why we applied the same range of dataset sizes as in previous research examining ALE of functional data.

**Fig. 1.**
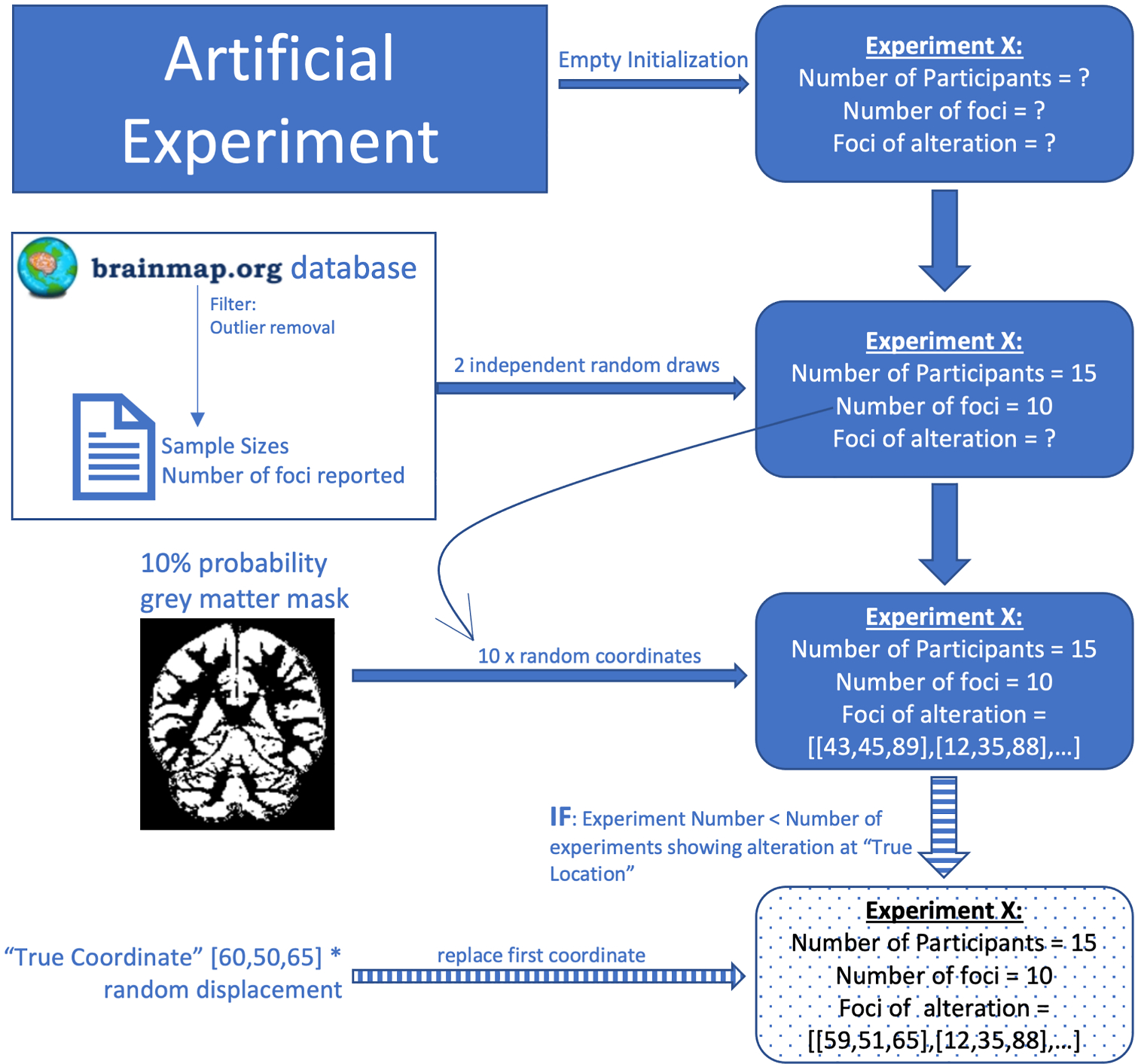
Simulation workflow. Two independent draws from the filtered structural Brainmap database were used to determine the sample size and the number of foci reported by the experiment. Next, we sampled the corresponding number of coordinates from a lenient gray-matter mask. Last, the first coordinate was replaced by the “true” coordinate multiplied with a displacement factor. This last step only occurred if the experiment was chosen to be an experiment showing alterations at the target location.

Each experiment’s relevant characteristics, namely number of participants and number of reported foci, were randomly drawn from distributions based on the structural neuroimaging part of the BrainMap database. Before sampling, we removed 17 experiments, which featured more than 500 participants, constituting extreme outliers (Z > 5). This left us with 4270 experiments in total. The coordinates reported by each experiment were uniformly sampled from a gray-matter mask created from the ICBM tissue probability maps (> 10% probability for gray matter; (Evans et al., 1994)). To model convergence in the datasets, we chose a ground-truth location in the left primary motor cortex (–30/–26/58 MNI space) and varied the number of experiments per analysis reporting a focus in the vicinity of this location from zero to ten. For these experiments, a coordinate that was based on the “true location” and then slightly displaced by a spread distribution (Eickhoff et al., 2016) replaced the first simulated random coordinate. The particular location of the ground-truth effect has no impact on the results of the study and was chosen only for visualization purposes. Varying both the total number of experiments and the number of experiments featuring a “true” alteration yielded a total of 330 unique combinations, for each of which we created 500 datasets to account for the randomness in the dataset simulation. This yielded a total of 165’000 datasets (Fig. 1). As a post-hoc addition to the contribution analysis, we additionally simulated 5000 smaller datasets comprising 5 to 15 experiments.

Real-life ALE meta-analyses are almost always looking at specific tasks, subject groups or individual disorders, which means that they could potentially feature more homogenous parameter distributions than our simulated datasets. For example, experiments on major depressive disorder might on average feature more subjects than experiments on schizophrenia, just based on prevalence alone (Pedersen et al., 2014). To rule out the potential that our findings relating to the excessive contribution of individual experiments were driven by the heterogeneity of our datasets, we ran supplementary analysis based on the 25%, 50% and 75% quantiles of the two important parameter distributions, namely number of subjects and number of foci. We created four categories for each parameter: 1. low, which meant sampling only up to the 25% quantile 2. low-medium, which meant sampling between the 25% and 50% quantile 3. high-medium, which meant sampling between the 50% and 75% quantile and 4. high which meant sampling above the 75% quantile (Table 3).

By combining each category from one parameter with all categories from the other parameter we got 16 groups (low/low, low/low-medium, low/high-medium, low/high, low-medium/low, etc.) for each of which we created 500 datasets per dataset size (number of experiments: 5–30) to account for the randomness in the sampling procedure. This yielded a total of 200000 datasets.

### 2.2. ALE analyses

We ran ALE meta-analyses using the latest implementation of the ALE algorithm (Eickhoff et al., 2016; Frahm et al., 2022; Turkeltaub et al., 2012). We then tested for statistically significant spatial convergence across experiments using voxel- and cluster-level thresholding (cluster-forming threshold at voxel-level p<.001), respectively, corrected for multiple comparisons by restricting the family-wise error (FWE) rate to p < .05. All analyses were run with a Python version of the ALE algorithm as used and described previously (Frahm et al., 2022) (https://github.com/LenFrahm/pyALE).

### 2.3. Outcome measures

After meta-analyzing all datasets, we evaluated the results obtained with FWE-corrected voxel-level or cluster-level thresholding (vFWE or cFWE) on three different outcome measures. First, we assessed each method’s sensitivity, which is given by the percentage of datasets for which the algorithm indicated a significant cluster in the close vicinity of the “true location”. This close vicinity was determined to be 4mm (or 2 voxels) in each direction, by exploratory analyses. When only counting datasets in which the “true location” was significantly altered we would end up underestimating the algorithms sensitivity, due to the displacement of the “true coordinate” moving around the center of the clusters. Obviously, high detection rates were desirable. Second, we looked at the susceptibility to spurious convergence, which is given by the rate of significant clusters outside the “true” location’s close vicinity. As these clusters are known to be the result of incidental convergence, a desirable thresholding method should keep this as low as possible. Third, we looked at the relative contribution of the most dominant experiments to the ALE values of voxels in spurious clusters and the impact of the thresholding methods on this number. The contribution was calculated by sorting the modeled activation values (Turkeltaub et al., 2012) per voxel in decreasing order, subtracting them from one, and calculating the cumulative product. This gave us the fraction of ALE value per voxel including only the most dominant experiment, only the two most dominant experiments, only the three most dominant experiments, etc. We then averaged these fractions over all voxels in the given cluster, which allowed us to assess the contribution of the most dominant experiments across the whole cluster. Following previous literature, any given cluster should not be driven by more than 50% by a single experiment (Eickhoff et al. 2016), which serves as a cut-off point for the lower bound recommendation. For this last outcome measure we additionally considered uncorrected inference, as a third way of significance thresholding.

## 3. Results

### 3.1. Differences between task and VBM database

The functional and structural BrainMap databases differed in their parameter distributions, reflecting typical differences in study design and outcomes between the two neuroimaging modalities (see Tables 1 and 2). Both the number of subjects per experiment and the number of foci each experiment reports are very influential in regard to the ALE algorithm outcome. The higher the number of subjects, the tighter the Gaussian probability distribution replacing the coordinates in the modeled activation maps. An experiment with a high number of participants has a relatively strong impact at the precise locations of its reported foci, while an experiment with only few participants has a much lower and more diffuse impact. The number of foci influences the amount of convergence observed by chance, and therefore strongly influences the thresholds resulting from vFWE and cFWE permutation testing. When comparing the structural and functional imaging subsets of the BrainMap database, we observed that on average structural neuroimaging experiments included many more participants than did experiments investigating task-related brain activations (independent samples t-test: *t* = *75.76, p*<*0.0001*). The number of foci reported was higher for task-activation (vs. VBM) experiments featuring more foci on average (medians: 6 vs 4; independent samples t-test: *t* = *92.14, p*<*0.0001*).

### 3.2. Sensitivity, susceptibility to spurious convergence, and cluster size

**Fig. 2.**
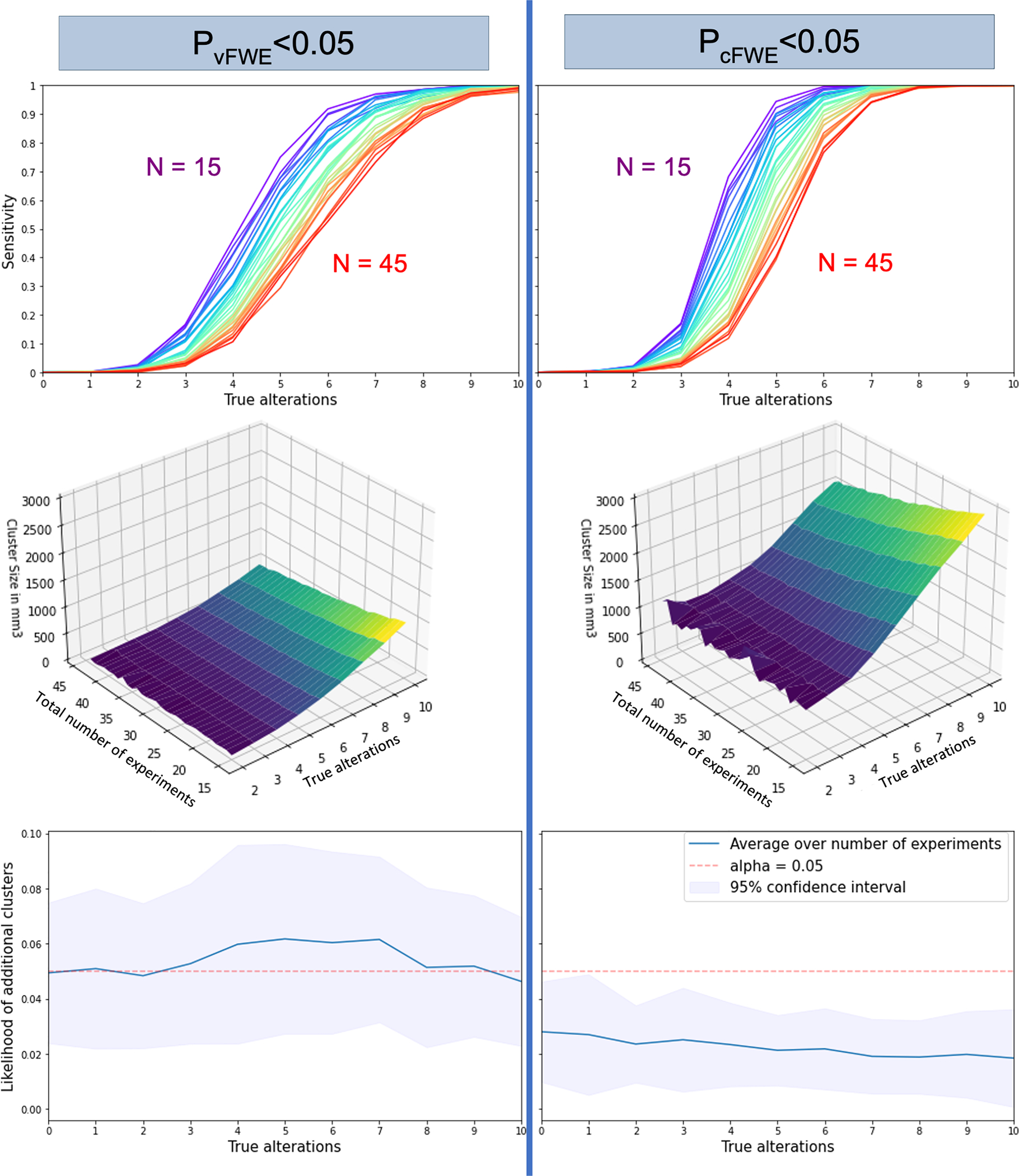
Comparing voxel- and cluster-level FWE on three outcome measures. Top: Sensitivity to detect clusters at the “true” location (4 mm radius). The x-axis represents the number of experiments that feature an alteration at the true location, while the y-axis represents the fraction of clusters which the algorithm recognizes as significant per parameter combination. Each total number of experiments has its own curve in the graph, following a spectral color sequence (15 purple - 45 red). Both graphs showcase curves of roughly sigmoid fashion, with clusters based on 1–3 alterations not being detected often, strong sensitivity gain around the 4–7 alteration mark, and almost perfect sensitivity over 8 alterations activating the target location. Overall, cFWE outperforms vFWE and features higher sensitivity at any stage. Middle: Size of significant clusters at the “true” location (4 mm radius). x- and y-axes indicate the total number of experiments and the number of experiments featuring a “true” alteration, respectively. The higher the rate of experiments featuring a true alteration compared to the total number, the larger the resulting clusters. cFWE correction yields much larger clusters than vFWE correction. Bottom: Rate of spurious clusters. Both multiple-comparison correction methods succeeded at controlling for an alpha error of .05.

Comparing the two significance thresholding approaches (vFWE and cFWE) regarding their sensitivity, susceptibility to spurious convergence, and the resulting cluster sizes, we found that our results served as a useful extension to Eickhoff et al.’s (2016) results (see Fig. 2). Even though the simulation parameters are fairly different between the studies, based on the differences between the databases used, ALE’s performance on the three outcome measures was very stable. Both vFWE and cFWE corrections control the susceptibility to spurious convergence to the expected degree, that is, corresponding to an alpha error level of 5%. cFWE achieved higher sensitivities than vFWE, while losing a bit of spatial specificity due to the higher cluster sizes it yields. These results indicate that the assumptions and recommendations about the ALE algorithm also hold when applied to VBM studies.

### 3.3. Excessive contribution of individual studies

Since Eickhoff and colleagues’ simulation study (2016), n = 17 experiments has been the recommended lower bound for dataset sizes when performing ALE analyses thresholded with cFWE (8 experiments for vFWE). For datasets larger than this minimum number, the dominant experiment’s average contribution to a significant cluster was found to be < 50%. We found that when dealing with VBM data, this lower bound becomes slightly higher. This results from two factors: 1. The lower number of foci reported by VBM experiments and 2. the higher average number of participants included in structural neuroimaging experiments, which in turn leads to tighter Gaussian distributions on average. When using cFWE-corrected thresholding, we observed that 23 experiments were required to ensure dominant contributions of less than 50%. This corresponds to an average contribution of around 80% by the two most dominant experiments. For the more conservative voxel-level FWE-corrected thresholding, around 14 experiments were sufficient to limit the average contribution of the most dominant experiment to 50%. At this point the two most dominant experiments contributed slightly less than 90% on average.

### 3.4. Contribution in homogeneous datasets

**Fig. 3.**
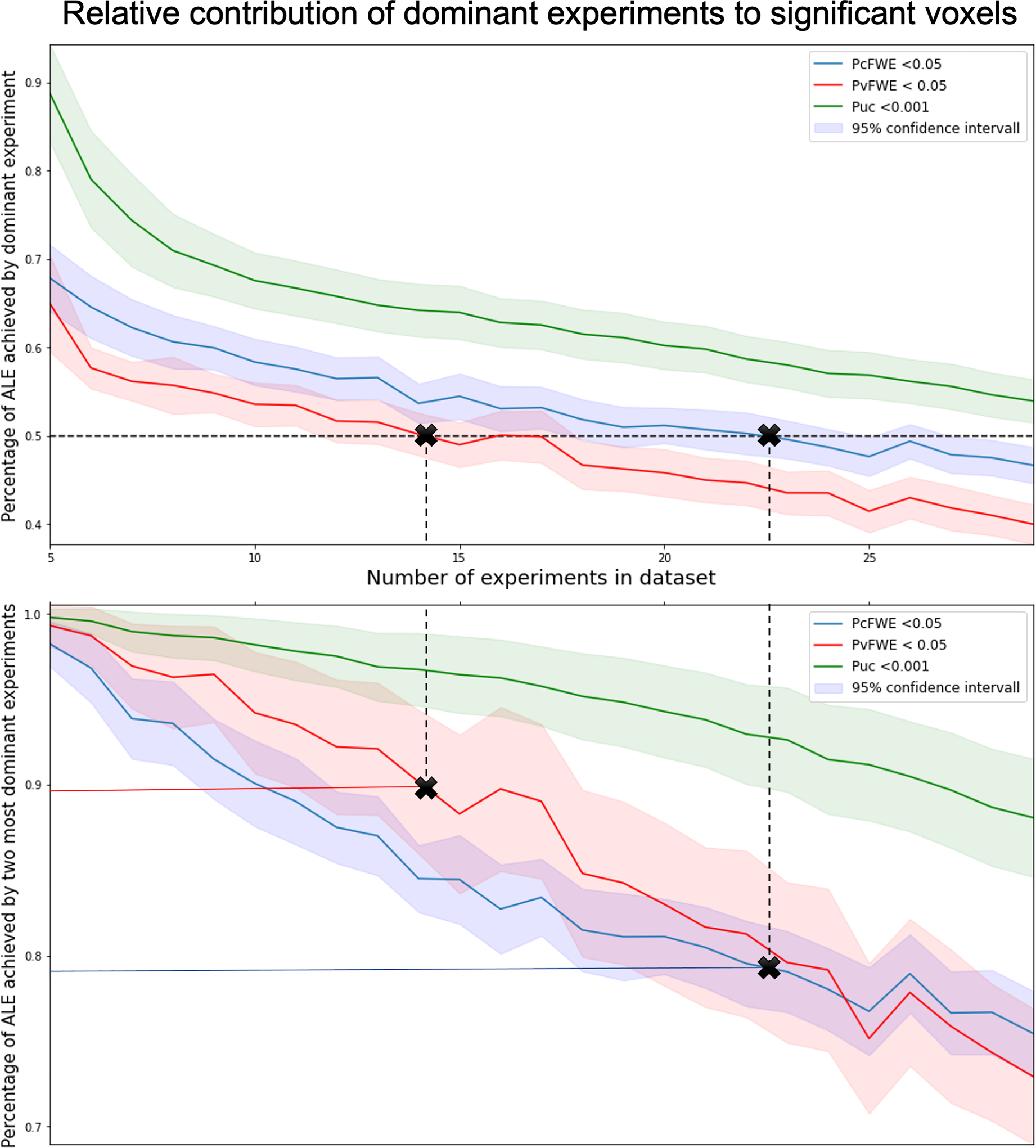
Quantification of the contribution of the single most (top) and two most (bottom) dominant experiments to spurious clusters. The smaller the ALE analysis dataset, the more likely resulting clusters are driven by single experiments. As ALE is supposed to find convergence across the literature, such excessive individual contributions need to be avoided, and any given dataset should include a minimum number of experiments. The figures show the fraction of ALE scores contributed by the most dominant experiments, averaged over all voxels in a given cluster. We compared three significance thresholding methods (uncorrected, vFWE-, cFWE-corrected), denoted by differently colored lines and corresponding confidence intervals in a lighter color of the same hue.

When looking at more homogenous datasets, where both the number of subjects and the number of foci were sampled from distribution spaces separated by the 25%, 50% and 75% quantile, we observe that the overall structure is very similar to what we find when sampling the complete VBM database. In fact, when averaging over all 16 groups the graphs become very similar to the ones in Fig. 3. Note that we here focus on cFWE as it is the current gold standard (Eickhoff et al., 2016) but the inferences made hold for vFWE as well.

**Fig. 4.**
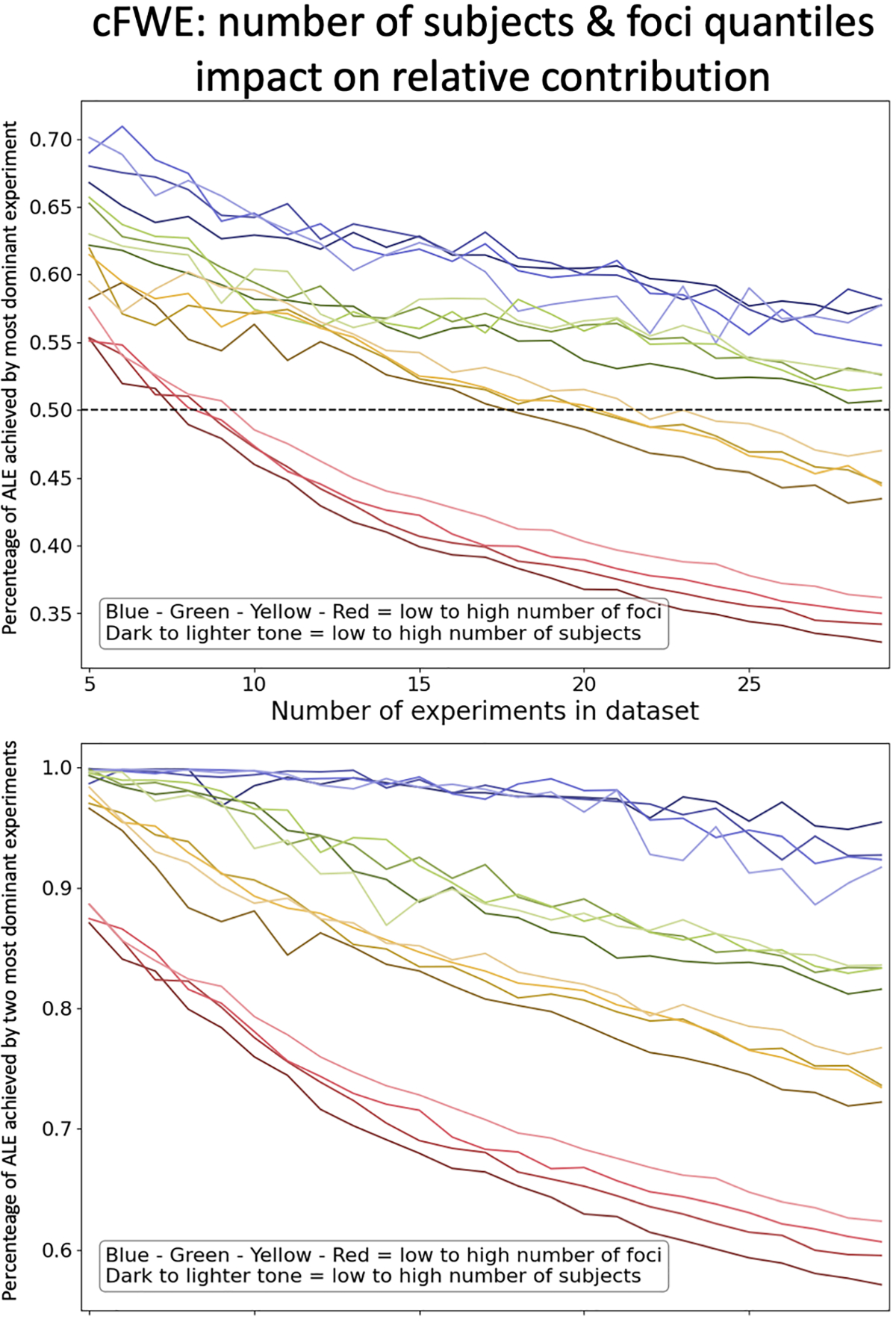
Relative contribution to significant clusters after correcting for multiple comparisons with cFWE. Quantification of the contribution of the single most (top) and two most (bottom) dominant experiments to spurious clusters. The figures show the fraction of ALE scores contributed by the most dominant experiments, averaged over all voxels in a given cluster The underlying datasets are created by sampling from specific quantiles of the number of subjects and foci distribution. Each line represents a certain sampling quantile combination. The four different colors indicate datasets sampling from low to high number of foci and the lightness/darkness of the color indicates sampling from low to high number of subjects.

When sampling over the whole VBM database 23 experiments were enough to ensure below 50% contribution by the most dominant experiment when correcting with cFWE. The quantile-based sampling employed here showed that this number might be too liberal or conservative depending on the characteristics of the individual meta-analysis dataset (Fig. 4). Datasets in which all experiments feature less than 5 foci (blue and green lines) for example do not reach below 50% contribution even if they include 29 experiments, which are the largest datasets we analyze in this analysis. There are two main relationships that influence the relative contribution. The first relationship is that the higher the average number of subjects in a dataset, the higher the average percentage of ALE achieved by the most dominant experiment. The second relationship is that the higher the average number of foci per experiment, the lower the dominant contribution. The effect the number of foci has on the relative contribution seems to be much stronger than the effect of the number of subjects.

## 4. Discussion

This study aimed to assess the stability of the ALE algorithm, significance thresholding, and sample size recommendations when dealing with structural neuroimaging (VBM) data. This work constitutes an extension of previous work (Frahm et al. 2022; Eickhoff et al. 2016), in which datasets were simulated by sampling from the functional (task-activation) BrainMap database. There are quite substantial differences between task-activation and VBM experiments in parameters that impact ALE outcomes substantially. On average, VBM experiments include many more participants and report fewer peaks than task-activation experiments. To assess ALE’s stability in the face of different average study characteristics, we simulated and meta-analyzed 365’000 datasets with different parameter combinations based on the VBM subset of the BrainMap database (Vanasse et al., 2018). We then evaluated them with respect to three outcome measures.

In general, the performance of the ALE algorithm was very stable across modalities, with the algorithm’s sensitivity and specificity reaching similar levels for functional and structural data. In particular, cFWE-corrected thresholding features higher sensitivity than vFWE-corrected thresholding in almost every scenario, and both techniques control the alpha error to the required and expected extent. Even though ALE has been applied to structural data for quite some time, this investigation serves as a systematic validation of VBM-ALE.

One difference we observed was that in structural ALE, single experiments had, on average, a higher impact towards significant clusters. The most important reason for this is the lower number of foci of alteration typically reported in VBM experiments. This sparseness of results leads to a very low cFWE threshold, as the assumption is that there is going to be very little to no convergence in random data. In this case very few converging studies will be enough to exceed the threshold which in turn leads to high average contribution per study. Another reason for the higher impact of single experiments towards significant clusters are the higher average number of subjects included in structural neuroimaging experiments and the resulting tighter Gaussian kernels used to model spatial uncertainty in ALE. As ALE is supposed to indicate convergence across a body of literature, care should be taken to ensure that significant clusters are not driven by single studies. The contribution of the most dominant experiment should therefore not exceed 50%. This is of course an arbitrary threshold, but it serves as a good approximation of what we would call “excessive contribution”, especially when looking at smaller datasets. Following the results of our analysis, we therefore recommend that researchers should aim to include at least 23 experiments in their VBM-ALE datasets. At that point, the average contribution of the dominant experiment reached the aforementioned 50% criterion when controlling for multiple comparisons via cFWE correction. This corresponds to an average contribution below 80% by the two most dominating experiments. It should be noted, though, that it is of course possible to run an ALE analysis on smaller datasets, no matter if they are functional or structural. These cases, however, require special diligence from the authors in assessing the contributions of experiments to individual clusters carefully. Conversely, even analyzing datasets with more than 23 experiments does not prevent clusters from being mainly driven by a small fraction of experiments in the dataset. As such, the recommended minimum number of n = 23 experiments for meta-analyses of VBM studies is not to be considered a hard cut-off and does not come with a guarantee of obtaining only broadly supported results, but it offers a heuristic as to what minimum breadth of support can be expected for the results.

An increasingly used approach to assessing the robustness of ALE results is running a jackknife analysis, which is a leave-one-out cross-validation scheme. This means recomputing n-1 (total number of experiments - 1) ALEs, always dropping another experiment from the dataset. The analysis allows ascertaining that clusters that reach significance in every fold of the cross-validation are not driven by a single experiment. While definitely improving on the conclusiveness of ALE results, the approach loses some of its information value at the interaction of two experiments driving the cluster. At this point, the cluster would appear significant in every fold of the cross-validation, but two experiments might still be a very miniscule fraction of the total number of experiments in the dataset. It appears questionable if such clusters should be counted as robust. Future research should investigate possibilities to better quantify and consider contributions and the robustness of clusters to assist researchers in the weighting and interpretation of their results.

These simulations were modeled following naturalistic parameter settings as closely as possible. It should be noted, however, that the parameters of any real dataset, especially regarding convergence, cannot be exactly known a priori. This is a limitation of any simulation work, which is why recommendations derived from such work should not be perceived as absolute. Specifically, when talking about the contribution and robustness of clusters, researchers should always examine their findings carefully. Another important factor not taken into account in this simulation work is the quality of the included studies, which has been identified as a major influencer of meta-analysis validity (Mueller et al., 2018; Tahmasian et al., 2019). Unfortunately, it is not possible to simulate experiment quality and it is therefore not in the scope of this research to evaluate the impact of study quality on ALE results. Furthermore, our simulation results cannot be extrapolated to other coordinate-based meta-analysis techniques, like multilevel kernel-density analysis (Kober and Wager, 2010; Nee et al., 2007; Wager et al., 2009) or seed-based d mapping (Albajes-Eizagirre et al., 2018; Radua et al., 2012; Radua et al., 2010). While the underlying core concept is shared between all of these techniques, the actual statistical implementation differs quite substantially. Even though there are some meta-analysis publication using, comparing and showing convergence between ALE and SDM (e.g., Enge et al., 2021), we feel that a more thorough and systematic comparison between all CBMA-methods would be an important step for the neuroimaging meta-analysis community.

## 5. Conclusion

We performed large-scale simulations based on parameters derived from the structural neuroimaging part of the BrainMap database to assess the behavior, power, and sensitivity of ALE when dealing with VBM data. Our investigation serves as the first formal validation of structural ALE, showcasing the algorithm’s stability when applied to coordinates derived from neuroimaging data of a different modality than evaluated in prior work. For conducting an ALE meta-analysis of structural neuroimaging experiments (i.e., VBM data), researchers should aim to be somewhat more conservative than recommended previously for task-activation studies, that is, they should aim to include 23 or more experiments.

## Figures and Tables

**Table 1:** Descriptive Statistics of the Number of Participants in Voxel-based Morphometry (VBM) and Task-Activation (TA) Subsets of the BrainMap Database

	VBM	TA

Min / Max	2 / 479	1 / 322
Mean / Median	30.63 / 20	14.69 / 12
Standard deviation	40.91	10.70

**Table 2:** Descriptive Statistics of the Number of Foci reported in Voxel-based Morphometry (VBM) and Task-Activation (TA) Subsets of the BrainMap Database

	VBM	TA

Min / Max	1 / 75	1 / 97
Mean / Median	6.54 / 4	8.27 / 6
Standard deviation	7.88	8.10

**Table 3:** Quantiles of the number of subjects & number of foci parameter distribution in the BrainMap VBM database

Quantile	Number of Subjects	Number of Foci

25%	13	2
50%	20	4
75%	32	8

## Data Availability

The code used to calculate the findings of this study is openly available at https://github.com/LenFrahm/VBM_ALE.
